# Early COVID-19 First-Dose Vaccination Coverage Among Residents and Staff Members of Skilled Nursing Facilities Participating in the Pharmacy Partnership for Long-Term Care Program — United States, December 2020–January 2021

**DOI:** 10.15585/mmwr.mm7005e2

**Published:** 2021-02-05

**Authors:** Radhika Gharpure, Angela Guo, Courtney Krier Bishnoi, Urvi Patel, David Gifford, Ashley Tippins, Aaron Jaffe, Evan Shulman, Nimalie Stone, Elisabeth Mungai, Suparna Bagchi, Jeneita Bell, Arjun Srinivasan, Anita Patel, Ruth Link-Gelles

**Affiliations:** ^1^CDC COVID-19 Response Team; ^2^American Health Care Association, Washington, DC; ^3^Palantir Technologies, Denver, Colorado; ^4^Centers for Medicare & Medicaid Services, Baltimore, Maryland; ^5^Division of Healthcare Quality Promotion, National Center for Emerging and Zoonotic Infectious Diseases, CDC.

Residents and staff members of long-term care facilities (LTCFs), because they live and work in congregate settings, are at increased risk for infection with SARS-CoV-2, the virus that causes coronavirus disease 2019 (COVID-19) (*1*,*2*). In particular, skilled nursing facilities (SNFs), LTCFs that provide skilled nursing care and rehabilitation services for persons with complex medical needs, have been documented settings of COVID-19 outbreaks (*3*). In addition, residents of LTCFs might be at increased risk for severe outcomes because of their advanced age or the presence of underlying chronic medical conditions (*4*). As a result, the Advisory Committee on Immunization Practices has recommended that residents and staff members of LTCFs be offered vaccination in the initial COVID-19 vaccine allocation phase (Phase 1a) in the United States (*5*). In December 2020, CDC launched the Pharmacy Partnership for Long-Term Care Program* to facilitate on-site vaccination of residents and staff members at enrolled LTCFs. To evaluate early receipt of vaccine during the first month of the program, the number of eligible residents and staff members in enrolled SNFs was estimated using resident census data from the National Healthcare Safety Network (NHSN^†^) and staffing data from the Centers for Medicare & Medicaid Services (CMS) Payroll-Based Journal.^§^ Among 11,460 SNFs with at least one vaccination clinic during the first month of the program (December 18, 2020–January 17, 2021), an estimated median of 77.8% of residents (interquartile range [IQR] = 61.3%– 93.1%) and a median of 37.5% (IQR = 23.2%– 56.8%) of staff members per facility received ≥1 dose of COVID-19 vaccine through the Pharmacy Partnership for Long-Term Care Program. The program achieved moderately high coverage among residents; however, continued development and implementation of focused communication and outreach strategies are needed to improve vaccination coverage among staff members in SNFs and other long-term care settings.

The Pharmacy Partnership for Long-Term Care Program is a public-private partnership among CDC, CVS Pharmacy (https://www.cvs.com), Managed Health Care Associates, Inc. (https://www.mhainc.com/home), and Walgreens (https://www.walgreens.com) to provide on-site COVID-19 vaccination of residents and staff members at enrolled LTCFs in 54 jurisdictions (49 states, four cities, and one territory).^¶^ These organizations report facility-level aggregate vaccine administration data to CDC through a web-based data platform. For this analysis, COVID-19 vaccine administration data were restricted to those from enrolled SNFs with a unique, valid CMS Certification Number (CCN) that had a vaccination clinic conducted on site during the first month of the program (December 18, 2020–January 17, 2021). The number of residents eligible for vaccination was estimated using the mean of NHSN weekly resident census counts for each facility during the weeks of December 14, 2020–January 17, 2021. Resident census data were available for 11,376 facilities; 60 (0.5%) facilities with missing data were excluded from analyses of resident vaccination, as were 24 (0.2%) facilities where the CCN was linked to NHSN reporting from multiple sites. The number of staff members eligible for vaccination was estimated using CMS Payroll-Based Journal counts of unique staff members for each facility during July–September (Quarter 3) 2020. Payroll data were available for 11,134 facilities; 326 (2.8%) facilities with missing data were excluded from analyses of staff member vaccination.

To estimate vaccination coverage, vaccine administration data for residents and staff members were matched to denominators for these groups using the facility CCN. National vaccination estimates included all CMS-certified SNFs with available denominator data and at least one on-site clinic in the first month of the program across all participating jurisdictions. Jurisdiction-level estimates are shown only for jurisdictions where >50 CMS-certified SNFs had at least one on-site clinic in the first month of the program and denominator data were available; data for participating cities were combined with those of their respective states for jurisdiction-level estimates. No individual-level data were included in the data files provided to CDC. All analyses were performed using SAS statistical software (version 9.4; SAS Institute). This activity was reviewed by CDC and was conducted consistent with applicable federal law and CDC policy.**

During December 18, 2020–January 17, 2021, among 12,702 CMS-certified SNFs enrolled in the Pharmacy Partnership for Long-Term Care Program, 11,460 (90.2%) had at least one on-site vaccination clinic conducted through the program.^††^ A total of 713,909 residents and 582,104 staff members received ≥1 COVID-19 vaccine doses.^§§^ Among 11,376 (99.3%) of these facilities with available resident census data, a median estimated 77.8% (IQR = 61.3%–93.1%) of residents were vaccinated; and among 11,134 (97.2%) facilities with available staff member payroll data, a median of 37.5% (IQR = 23.2%–56.8%) of staff members were vaccinated (Figure 1). Among the 54 participating jurisdictions, 40 states had >50 CMS-certified SNFs that conducted at least one on-site clinic during the first month of the program and had available denominator data; the median percentage of residents vaccinated by state ranged from 65.7% to >100%^¶¶^ and of staff members, ranged from 19.4% to 67.4% (Figure 2).

**FIGURE 1:**
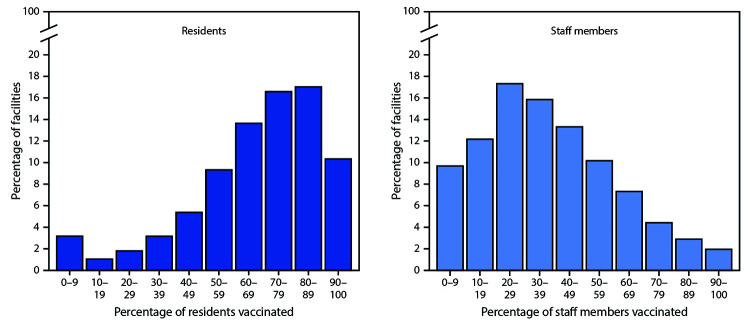
Estimated percentage* of residents^†^ and staff members^§^ at skilled nursing facilities^¶^ enrolled in the Pharmacy Partnership for Long-Term Care Program who received ≥1 dose of COVID-19 vaccine — United States, December 18, 2020–January 17, 2021 **Abbreviations:** CMS = Centers for Medicare & Medicaid Services; COVID-19 = coronavirus disease 2019. * Vaccination coverage >100% (not shown) was estimated for residents in 2,118 (18.5%) facilities and for staff members in 559 (4.8%) facilities. Estimated vaccination coverage in excess of 100% might reflect resident and staff member turnover, other variation in denominator estimates, or errors in reported vaccine administration data. ^†^ n = 11,376 facilities. The number of residents eligible for vaccination was estimated using the mean of National Healthcare Safety Network weekly resident census counts for each facility during December 14, 2020–January 17, 2021. ^§^ n = 11,134 facilities. The number of staff members eligible for vaccination was estimated using CMS Payroll-Based Journal counts of unique staff members for each facility during July–September (Quarter 3) 2020. Vaccination estimates reflect staff members vaccinated through the Pharmacy Partnership for Long-Term Care Program; additional staff members might have been vaccinated through other programs. ^¶^ Includes facilities with a unique, valid CMS Certification Number and with at least one on-site clinic conducted through the Pharmacy Partnership for Long-Term Care Program during December 18, 2020–January 17, 2021.

**FIGURE 2:**
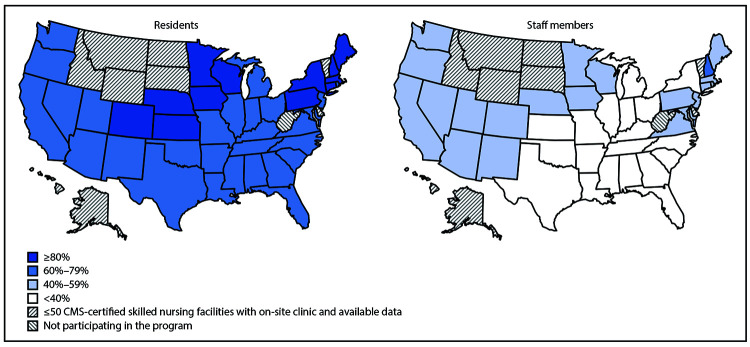
Estimated median percentage of residents* and staff members^†^ at skilled nursing facilities^§^ enrolled in the Pharmacy Partnership for Long-Term Care Program who received ≥1 dose of COVID-19 vaccine, by jurisdiction^¶^ — United States, December 18, 2020–January 17, 2021 **Abbreviations:** CMS = Centers for Medicare & Medicaid Services; COVID-19 = coronavirus disease 2019. * n = 11,376 facilities. The number of residents eligible for vaccination was estimated using the mean of National Healthcare Safety Network weekly resident census counts for each facility during December 14, 2020–January 17, 2021. ^†^ n = 11,134 facilities. The number of staff members eligible for vaccination was estimated using CMS Payroll-Based Journal counts of unique staff members for each facility during July–September (Quarter 3) 2020. Vaccination estimates reflect staff members vaccinated through the Pharmacy Partnership for Long-Term Care Program; additional staff members might have been vaccinated through other programs. ^§^ Includes facilities with a unique, valid CMS Certification Number and with at least one on-site clinic conducted through the Pharmacy Partnership for Long-Term Care Program during December 18, 2020–January 17, 2021. ^¶^ Participating jurisdictions do not include West Virginia. Jurisdiction-level estimates are only presented for 40 states that had >50 CMS-certified skilled nursing facilities with a vaccination clinic conducted during December 18, 2020–January 17, 2021. Data for Chicago, New York City, and Philadelphia were combined with those of their respective states for jurisdiction-level estimates. Washington, DC, and Puerto Rico had ≤50 skilled nursing facilities with an on-site clinic and available data and are not shown.

## Discussion

The Pharmacy Partnership for Long-Term Care Program partners with pharmacy providers to manage the COVID-19 vaccination process, reducing the workload for SNF administrators and jurisdictional health departments by coordinating scheduling, vaccine cold chain management, patient counseling, and vaccine administration. In the first month of the program, more than one million SNF residents and staff members in CMS-certified SNFs received on-site COVID-19 vaccination, with moderately high coverage among residents. Considering the high COVID-19–associated morbidity and mortality in SNFs (*1*,*2*) and, particularly, the risk for severe disease among SNF residents (*3*), vaccination of this population is a public health priority. However, the lower percentage of staff members vaccinated raises concern about low coverage among a population at high risk for occupational exposure to SARS-CoV-2.

Low vaccination coverage among staff members working in LTCFs has been previously described for influenza vaccination; during the 2017–18 influenza season, vaccination coverage among LTCF staff members was lower than that among other health care workers (*6*), and survey data suggest that hesitancy among this population could be associated with skepticism about influenza vaccine effectiveness and perceived low risk for virus transmission to themselves or others (*7*). Although efforts are ongoing to promote confidence in COVID-19 vaccination among health care workers, challenges persist. According to a survey conducted in October 2020, 37% of nurses stated that they were not confident that a COVID-19 vaccine would be safe and effective, and only 34% agreed that they would voluntarily receive a COVID-19 vaccine.*** Frequently cited reasons for vaccine hesitancy included the perceived rapidity of vaccine development; inadequate information received about vaccine safety, side effects, and administration; and skepticism regarding the clinical trials and vaccine approval processes. Similarly, survey data from December 2020 indicated that nearly one third (29%) of respondents who worked in a health care delivery setting expressed COVID-19 vaccine hesitancy, and updated estimates from January 2021 indicated that hesitancy persisted, with 28% of health care workers indicating a desire to delay receipt of vaccine until they had more information about safety and effectiveness.^†††^ Specifically among LTCF staff members, a November 2020 survey found that only 45% of respondents were willing to receive a COVID-19 vaccine immediately once available, and an additional 24% would consider it in the future; the most frequently identified reason for vaccine hesitancy was concern about side effects (*8*). High staff member turnover, staff members working in multiple facilities (*9*), and limited resources for staff member outreach and education (*10*) are also potential barriers to vaccination in LTCFs. Use of focused communication messages to increase COVID-19 vaccine confidence in health care personnel^§§§^ and specifically among LTCF staff members^¶¶¶^, including messages regarding the documented safety and efficacy of authorized COVID-19 vaccines, might help improve vaccination acceptance and coverage. Staff members serve as a trusted source of information for patients and residents; therefore particularly in LTCF settings where residents and staff members might be vaccinated simultaneously, increasing vaccine confidence among staff members might have additional benefits for promoting vaccination among residents. Because coverage varied among jurisdictions, lessons learned from jurisdictions or individual facilities with high coverage might provide insight into strategies that could be applied more broadly.

The findings in this report are subject to at least four limitations. First, vaccination procedures for health care workers might have underestimated the percentage of staff members vaccinated. Some jurisdictions encouraged LTCF staff members to be vaccinated through other programs for health care worker vaccination (e.g., clinics conducted by health departments or hospitals); only staff members vaccinated on site through the Pharmacy Partnership for Long-Term Care Program were included in these staff member vaccination estimates. Allocations to pharmacies included adequate vaccine to cover all expected residents and staff members in each facility; however, vaccination of staff members might have been intentionally staggered by SNFs in accordance with CDC’s clinical considerations for health care providers, although staggering is emphasized for second doses in the 2-dose series.**** Similarly, scheduling of clinics could have posed challenges for staff members who worked on a shift schedule or worked at multiple facilities, or staff members might not have been available for vaccination around holidays falling within the time frame evaluated. Systematic data concerning these potential barriers were not recorded, and they require further study. Second, the number of residents and staff members eligible for vaccination at each facility was estimated using secondary data sources and was not determined in real time at each vaccination clinic. The most recent available CMS Payroll-Based Journal data were from July to September 2020 and might have differed from staffing during the time of vaccination clinics. Additional variation in facility occupancy and resident and staff member turnover during December 2020–January 2021 could affect the accuracy and precision of these denominator estimates. Third, these estimates only evaluated the first month of the program; vaccination coverage might have increased as subsequent clinics were conducted at each facility. Vaccination was only evaluated among CMS-certified SNFs because of the ability to match to secondary data sources using the facility CCN; these estimates might not be generalizable to all other LTCFs enrolled in the program (e.g., assisted living facilities and non-CMS certified facilities). Finally, no qualitative data were collected to determine motivators for vaccination or to document and characterize possible vaccine hesitancy suggested by the low percentage of staff members vaccinated.

Data on COVID-19 vaccine administration and coverage are essential to evaluating and supporting vaccination efforts over time. Additional data collected for the duration of the Pharmacy Partnership for Long-Term Care Program will characterize the percentage of residents and staff members vaccinated over time, as well as the percentage who complete the 2-dose series. Vaccine administration data can also be used to assess the effects of vaccination on COVID-19 case rates and transmission in high-risk settings; additional data will be collected through the NHSN LTCF Component.^††††^ Communications resources developed to increase vaccine confidence among LTCF staff members can be employed for public health outreach, and strategies to address structural barriers, such as scheduling around shift work or provision of paid medical leave for possible postvaccination side effects, should be encouraged. Further studies should explore differential vaccination coverage by characteristics, including geographic location, sociodemographic factors, and facility size, as well as characterize barriers to vaccination of persons working in LTCFs; qualitative assessment of attitudes and beliefs might inform additional communication strategies to improve vaccine confidence and increase vaccination among LTCF staff members.

**Summary**

**What is already known about this topic?**

Residents and staff members in long-term care facilities, particularly skilled nursing facilities (SNFs), are at increased risk for COVID-19–associated morbidity and mortality and have been prioritized for the first phase of vaccination in the United States.

**What is added by this report?**

Among 11,460 SNFs with at least one vaccination clinic conducted during the first month of the CDC Pharmacy Partnership for Long-Term Care Program, a median of 77.8% of residents and 37.5% of staff members received ≥1 vaccine dose through the program.

**What are the implications for public health practice?**

Barriers to SNF staff member vaccination need to be overcome with continued development and implementation of focused communication and outreach strategies to improve vaccination coverage.

## Footnotes

*
https://www.cdc.gov/vaccines/covid-19/long-term-care/pharmacy-partnerships.html

†
https://www.cdc.gov/nhsn/index.html

§
https://www.cms.gov/Medicare/Quality-Initiatives-Patient-Assessment-Instruments/NursingHomeQualityInits/Staffing-Data-Submission-PBJ

¶ Participating jurisdictions do not include West Virginia. Participating cities and territories include Chicago, Illinois; New York City, New York; Philadelphia, Pennsylvania; Washington, DC; and Puerto Rico.

** 45 C.F.R. part 46, 21 C.F.R. part 56; 42 U.S.C. Sect. 241(d); 5 U.S.C. Sect. 552a; 44 U.S.C. Sect. 3501 et seq.

†† Data reported to CDC as of January 24, 2021.

§§ Both Pfizer-BioNTech COVID-19 vaccine and Moderna COVID-19 vaccine were used in the Pharmacy Partnership for Long-Term Care Program; jurisdictional health departments designated one vaccine product for use in all enrolled facilities in their jurisdiction. Administration of second doses was not evaluated in this analysis.

¶¶ Estimated vaccination >100% might reflect resident and staff member turnover, other variation in denominator estimates, or errors in reported vaccine administration data.

***
https://www.nursingworld.org/practice-policy/work-environment/health-safety/disaster-preparedness/coronavirus/what-you-need-to-know/covid-19-vaccine-survey

††† https://www.kff.org/coronavirus-covid-19/report/kff-covid-19-vaccine-monitor-december-2020/; https://www.kff.org/coronavirus-covid-19/report/kff-covid-19-vaccine-monitor-january-2021/

§§§
https://www.cdc.gov/vaccines/covid-19/health-systems-communication-toolkit.html

¶¶¶
https://www.cdc.gov/vaccines/covid-19/toolkits/long-term-care/index.html

****
https://www.cdc.gov/vaccines/hcp/acip-recs/vacc-specific/covid-19/clinical-considerations.html

††††
https://www.cdc.gov/nhsn/ltc/index.html
